# Diffuse Reduction of Spleen Density Is an Independent Predictor of Post-Operative Outcomes After Curative Gastrectomy in Gastric Cancer: A Multi-Center Study

**DOI:** 10.3389/fonc.2020.01050

**Published:** 2020-06-30

**Authors:** Yun-Shi Huang, Xiao-Dong Chen, Ming-Ming Shi, Li-Bin Xu, Su-Jun Wang, Wei-Sheng Chen, Guan-Bao Zhu, Wei-Teng Zhang, Xian Shen

**Affiliations:** ^1^Department of Gastrointestinal Surgery, The Second Affiliated Hospital, Wenzhou Medical University, Wenzhou, China; ^2^Department of Gastrointestinal Surgery, The First Affiliated Hospital, Wenzhou Medical University, Wenzhou, China

**Keywords:** gastric cancer, spleen density, computed tomography, post-operative outcomes, prognostic roles

## Abstract

**Objectives:** The present study aimed to explore the association between spleen density and post-operative outcomes of patients after curative gastrectomy.

**Methods:** From June 2014 to December 2015, we conducted a retrospective study to analyze pertinent clinical data from gastric cancer patients who underwent gastrectomy at the First and the Second Affiliated Hospital of Wenzhou Medical University. Spleen density was determined via computed tomography scans. Univariate and multivariate analyses were performed to determine the risk factors associated with post-operative outcomes after gastric cancer surgery.

**Results:** Three hundred and ninety five patients were included, of whom 98 (24.8%) were defined as having a diffuse reduction of spleen density based on diagnostic cutoff values (spleen density ≤43.89 HU). Multivariate analysis revealed diffuse reduction of spleen density as an independent risk factor for post-operative complications and long-term overall survival.

**Conclusions:** Spleen density can predict severe postoperative complications and long-term overall survival in gastric cancer patients. As an imaging evaluation method, spleen density is a novel tool can be used in clinical as a prognostic predictor for patients with gastric cancer.

## Introduction

Gastric cancer remains the fifth most frequent malignancy and the third leading cause of cancer deaths globally (1). In China, 679,100 individuals were diagnosed with gastric cancer, and 498,000 gastric cancer-related deaths occurred in 2015 (2). Despite the development of multimodal treatments including surgery, traditional chemotherapy, and the implementation of neoadjuvant therapy, which can greatly improve the prognosis, gastric cancer is still a deadly disease with a poor clinical outcome (3). However, an accurate method to predict the prognosis of gastric cancer patients simply and effectively is not yet available.

The spleen is the largest peripheral immune organ and participates in the regulation of immune homoeostasis, but its role has been ignored among clinicians (4, 5). Diffuse reduction of spleen density (DROSD) is an imaging manifestation in the abdominal computed tomography (CT), which was originally reported in patients with acute pancreatitis (AP) (6). Meanwhile, in our long-term clinical practice, we also observed that this phenomenon existed in some gastric cancer patients. Interestingly, the post-operative prognosis of these patients was more serious. Some scholars found that AP patients with DROSD had more severe immune dysfunction than without (7). The host immune system is relevant to cancer development and progression (8, 9). Currently, there is a paucity of studies investigating the impact of DROSD in gastric cancer patients.

This study aimed to investigate whether DROSD, as determined by decreased CT values of the spleen, would predict post-operative outcomes in a cohort of patients after curative gastrectomy for gastric cancer.

## Methods

### Study Patients

**Figure 1:**
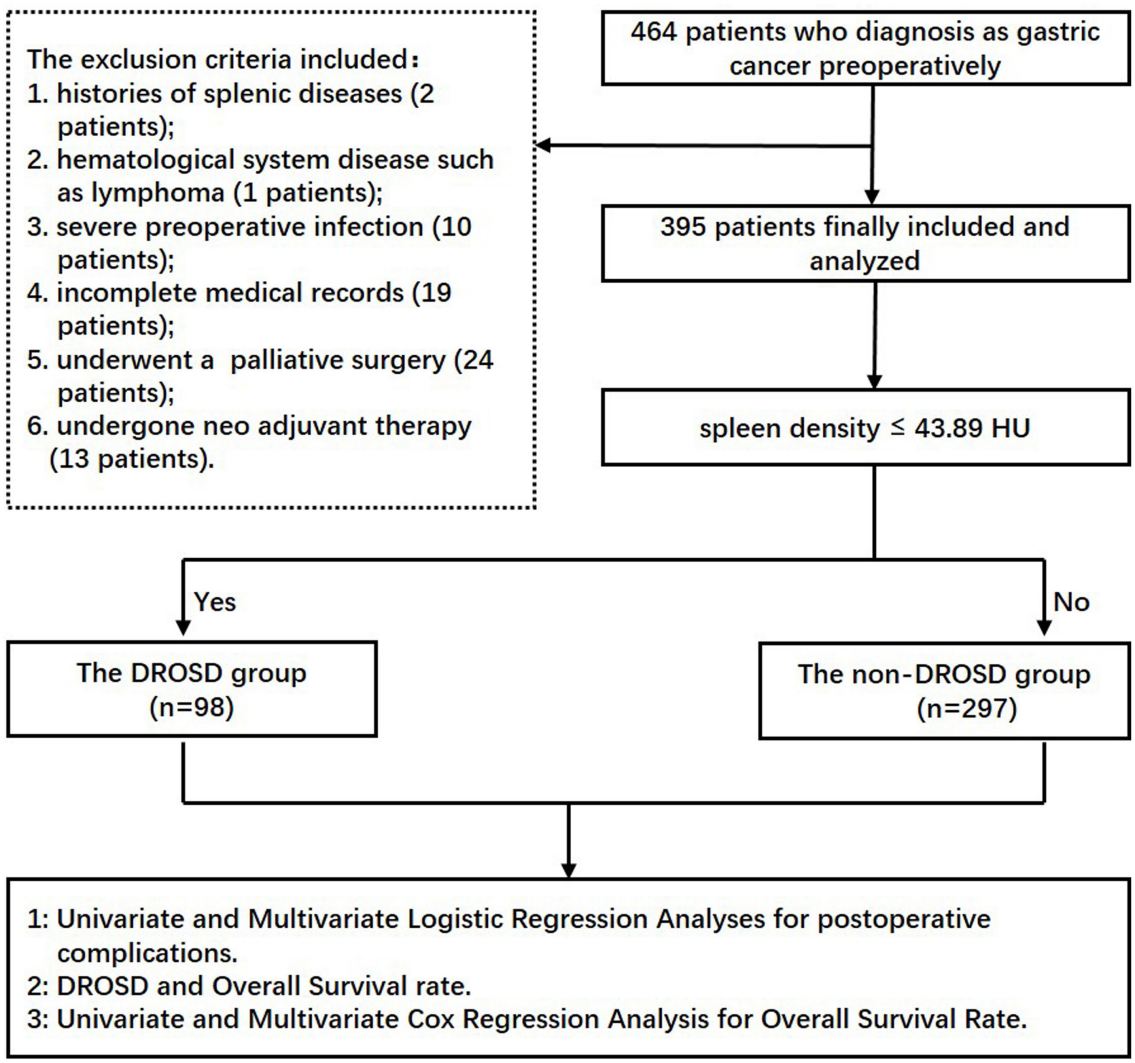
Block flow chart of experimental grouping.

This retrospective study was approved by the Ethics Committee of the First Affiliated Hospital of Wenzhou Medical University. From June 2014 to December 2015, only patients who underwent curative gastrectomy for gastric cancer were included, with the following criteria: (1) patients who underwent preoperative abdominal CT scans and had a serological examination within 1 month before surgery and (2) those who are willing to participate in this study and provide informed consent. The following patients were excluded: (1) patients with past histories of splenic diseases, (2) those who have hematological system disease such as lymphoma, (3) those who have severe preoperative infection, (4) those who have incomplete medical records, (5) those who underwent a palliative surgery, and (6) those who undergone neoadjuvant chemotherapy. We collected and analyzed the data from the remaining 395 GC patients who underwent radical gastrectomy in the First Affiliated Hospital of Wenzhou Medical University and the Second Affiliated Hospital of Wenzhou Medical University. The experimental flow chart is shown in Figure 1. All patients underwent conventional therapy following the Japanese Gastric Cancer Treatment Guidelines (10).

### Data Collection

We prospectively collected and analyzed the following data in this study: (1) age; gender; body mass index (BMI); Nutritional Risk Screening 2002 (NRS 2002) scores; albumin and hemoglobin concentration; platelet/lymphocyte ratio (PLR); neutrophil/lymphocyte ratio (NLR); sarcopenia; Charlson Comorbidity Index; the American Society of Anaesthesiologists (ASA) grade; hypertension; diabetes mellitus; previous abdominal surgery; differentiation of tumors; tumor location, size and TNM stage (preoperative patients and disease characteristics aspect); (2) laparoscopy-assisted, type of resection, combined resection, and type of reconstruction (the operative details aspect); and (3) long-term overall survival (any causes of deaths), postoperative complications and readmission within 1 month after surgery (the postoperative outcomes aspect).

### Measurement of the Spleen Density

**Figure 2:**
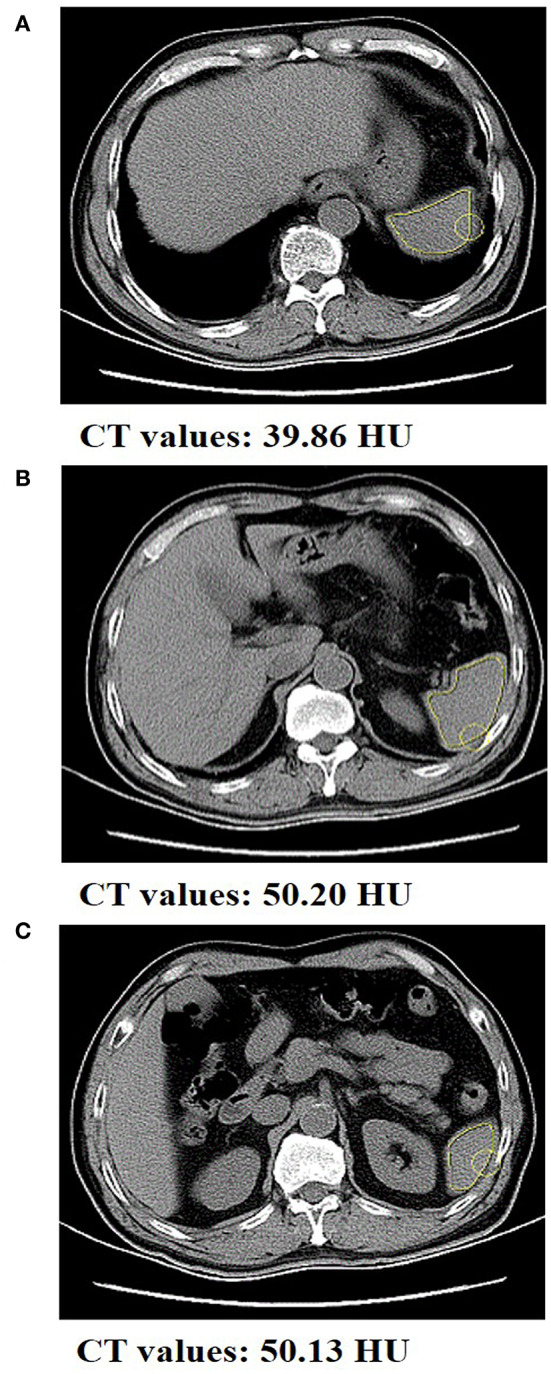
Abdominal CT scan of a gastric cancer patient with spleen density. The CT value of spleen at the **(A)** upper pole level, **(B)** the hilum level, and the **(C)** inferior pole level was 39.86, 50.20, and 50.13 HU, respectively.

Spleen densities of patients with gastric cancer were measured at the Department of Radiology of the First Affiliated Hospital of Wenzhou Medical University using the same scanning parameters. In short, location conditions included 120 kV of the tube voltage, 50 mA of the tube current, 750 ms of tube circumrotation time, 5 mm of the layer thickness, and 5 mm of the layer spacing. Retrospective analysis of non-enhanced CT scan sequence images was performed by two investigators. As shown in Figure 2, considering that the spleen density is affected by its own hemoperfusion, spleen CT values (Hounsfield units, HU) were measured at the upper pole, hilum, and inferior pole levels using a dedicated processing system (version 3.0.11.3 BN17 32 bit; INFINITT Healthcare Co. Ltd.). Spleen density was defined as the average of the three measurements of spleen CT values. In order to control for systematic errors, re-averaging of the spleen density measurements by the two investigators was ultimately performed. The mean of spleen density in this cohort were 46.95 HU (interquartile range, 43.91–49.80 HU).

The restrictive cubic splines by R Language was plotted to check whether spleen density is a linear risk structure. As shown in Supplementary Figure 1, the risk curve fluctuation obviously as spleen density gradually increases, considering the linear relationship is not very strong (*P* = 0.169). Therefore, categorical variable instead of continuous variable was used to analyze spleen density. To determine the spleen density cutoff values, with a mostly significant difference, we used optimum stratification to find the most significant *P*-value by means of log-rank chi-squared statistics (11). In the previous literature, this method has been presented to solve the threshold value of the continuous variable at which patients are best separated with respect to time to mortality (12). The cut-off values obtained by this method were used to classify patients into DROSD and non-DROSD.

### Follow-Up

All patients were required to come back and undergo the necessary examinations within the first month after surgery. After that, they were followed up every 3 months for further examinations as needed. Patients were contacted by phone and were scheduled to come back to the hospital to fulfill the follow-up program in the above time points. The follow-up program was consisted of a physical examination, laboratory tests, and ultrasonography and/or CT and/or endoscopy. Data on patient mortality, including time and cause of death, were obtained primarily through medical records, telephone follow-up and the local population database. Overall survival rate was determined as the proportion of all patients who survive after surgery for gastric cancer. Postoperative complication was recorded within a month after surgery, which was classified using the Clavien–Dindo (CD) Classification (13). Severe postoperative complications (SPCs) were defined as complications classified as Grade III or above. The date of the last follow-up was October 2018.

In this cohort, most of the patients were followed up by review of medical records and phone, and a small number of patients were contacted directly by phone. We eventually completed the follow-up work by retrieving data from the local population database for 29 patients who cannot be contacted by phone.

### Statistical Analysis

The Kolmogorov–Smirnov test was used to determine whether continuous data conform to normal distribution. Normal distribution, non-normal distribution, and categorical variables were presented as mean and standard deviation, median and interquartile range, and quantity and percentage, respectively. The Student's *t*-test was used to compare the data that conform to normal distribution. Mann–Whitney U-test was used to compare non-normally distributed data and Pearson chi-squared test or Fisher's exact test to compare categorical data. The outcome of this study was overall survival, calculated from the date of surgery to the date of death or last available follow-up. The Kaplan–Meier method was used to analyze the overall survival, and log-rank test was performed to compare the difference in survival between the subgroups. Multivariate Cox proportional hazards regression analysis was performed to determine the independent risk factors for long-term overall survival. Variables with *P* < 0.10 in a univariate analysis were included in the multivariate analysis, and variables with *P* < 0.05 were retained ultimately in the multivariate model.

All tests were two-tailed and were considered to be statistically significant when *P* < *0.05*. All data were analyzed using the SPSS Statistics version 20.0 (IBM, Armonk, New York, USA).

## Results

### Baseline Characteristics

From June 2014 to December 2015, a total of 395 patients met our criteria and were included for analysis. Demographic and clinical characteristics of patients with gastric cancer are represented in Table 1.

**Table 1:** Patient demographic and clinical characteristics.

**Factors**	**Total (*n =* 395)**	**DROSD (*n =* 98)**	**non-DROSD (*n =* 297)**	***P*-values**
Age, median, years*	65 (58–73)	66 (60–73)	64 (57–73)	0.735
Gender				0.799
Male	302 (76.5)	74 (75.5)	228 (76.8)	
Female	93 (23.5)	24 (45.5)	69 (23.2)	
BMI (kg/cm2)*	22.17 (20.21–24.22)	22.83 (20.47–24.61)	22.07 (20.20–24.03)	0.095
NRS score				0.763
1–2	242 (61.3)	62 (63.3)	180 (60.6)	
3–4	122 (30.9)	28 (28.6)	94 (31.6)	
5–6	31 (7.8)	8 (8.1)	23 (7.7)	
Preoperative albumin (g/L)*	37.9 (34.6–40.9)	36.9 (33.8–39.8)	38.6 (35.0–41.3)	0.405
Preoperative hemoglobin (g/L)*	123.5 (105.0–136.0)	112.0 (90.0–131.5)	126.0 (111.0–137.0)	<0.001‡
PLR*	149.7 (114.2–205.8)	163.5 (121.9–228.6)	142.4 (109.4–200.8)	0.196
NLR*	2.27 (1.74–3.24)	2.62 (2.02–3.56)	2.18 (1.66–3.11)	0.013‡
Sarcopenia				0.094
No	310 (78.5)	71 (72.4)	239 (80.5)	
Yes	85 (21.5)	27 (27.6)	58 (19.5)	
Charlson comorbidity index				0.058
0	203 (51.4)	42 (42.9)	161 (54.2)	
1–2	179 (45.3)	52 (53.1)	127 (42.8)	
3–6	13 (3.3)	4 (4.0)	9 (3.0)	
ASA score				0.098
1–2	317 (80.3)	73 (76.0)	244 (82.2)	
3–4	78 (19.7)	25 (24.0)	53 (17.8)	
Hypertension				0.017‡
No	294 (74.4)	64 (65.3)	230 (77.4)	
Yes	101 (25.6)	34 (34.7)	67 (22.6)	
Diabetes Mellitus				0.208
No	345 (87.3)	82 (83.7)	263 (88.6)	
Yes	50 (12.7)	16 (16.3)	34 (11.4)	
Previous abdominal surgery				0.179
No	343 (86.8)	89 (90.8)	254 (85.5)	
Yes	52 (13.2)	9 (9.2)	43 (14.5)	
Differentiated Types				0.993
Undifferentiated	29 (7.3)	6 (6.1)	23 (7.7)	
Differentiated	300 (75.9)	75 (76.5)	225 (75.8)	
Signet-ring cell	66 (16.8)	17 (17.4)	49 (16.5)	
Tumor site				0.401
Cardia	49 (12.4)	9 (9.2)	40 (13.4)	
Gastric body	77 (19.5)	21 (21.4)	56 (18.7)	
Antrum	253 (64.0)	63 (64.3)	190 (64.2)	
Diffuse	16 (4.1)	5 (5.1)	11 (3.7)	
Tumor size (cm)*	4.00 (2.00–5.50)	5.00 (2.75–6.00)	3.50 (2.00–5.00)	0.005‡
TNM stages				0.095
I	113 (28.6)	22 (22.4)	91 (30.6)	
II	78 (19.7)	19 (19.4)	59 (19.9)	
III	204 (51.6)	57 (58.2)	147 (49.5)	
Total gastric resection				0.467
No	242 (61.3)	57 (58.2)	185 (62.3)	
Yes	153 (38.7)	41 (41.8)	112 (37.7)	
Combined resection				0.467
No	358 (90.6)	87 (88.8)	271 (91.2)	
Yes	37 (9.4)	11 (11.2)	26 (8.8)	
Laparoscopy-assisted surgery				0.438
No	317 (80.3)	76 (77.6)	241 (81.8)	
Yes	78 (19.7)	22 (22.4)	56 (18.9)	
Type of reconstruction				0.620
Roux-en-Y	173 (43.8)	45 (45.9)	128 (43.1)	
Billroth I	149 (37.7)	36 (36.7)	113 (38.0)	
Billroth II	73 (18.5)	17 (17.4)	56 (18.9)	
C-D classification				0.030‡
≤ 1	302 (76.5)	67 (68.4)	235 (79.1)	
≥2	93 (23.5)	31 (31.6)	62 (20.9)	
Readmission of 30 days				0.765
No	364 (92.2)	91 (92.9)	273 (91.9)	
Yes	31 (7.8)	7 (7.1)	24 (8.1)	

*BMI, body mass index; NRS, nutritional risk screening; PLR, platelet/lymphocyte ratio; NLR, neutrophil/lymphocyte ratio; ASA, American Society of Anaesthesiologists; diffuse, linitis plastic; TNM, tumor-node-metastasis; C-D complication classification, Clavien-Dindo complication classification*.

*Values are number of patients and percent unless indicated otherwise*;

* *Values are median (inter quartile range)*;

‡ *Values are statistically significant (P < 0.05)*.

### Cutoff Values for Diffuse Reduction of Spleen Density (DROSD)

Cutoff value for DROSD associated with long-term overall survival was 43.89 HU. Using this cutoff value, 98 (24.8%) patients were found to have DROSD. As shown in Table 1, patients with DROSD had a lower preoperative hemoglobin level, higher NLR, hypertension, larger tumor size and poorer C–D Classification than those without DROSD (all *P* < 0.05).

### Actual Number and Frequency of Each Complication

As Table 2 shows, there were 157 postoperative events involving 93 patients (23.5%). Of them, 42 (10.6%) patients had grade IIIa or higher PCs. Pulmonary complications, which mainly included pulmonary infections and pleural effusions, and intra-abdominal infections were the most frequent PCs. Postoperative infection complications were included Grade II or above wound infection, pulmonary infections and intra-abdominal infections.

**Table 2:** Actual number and frequency of each complication (Grade ≥ II).

**Complication**	**Total (*n =* 395)**	**DROSD (*n =* 98)**	**non-DROSD (*n =* 297)**	***P*-values**
Wound infection	3 (0.8)	1 (1.0)	2 (0.7)	>0.05
Intra-abdominal infection	34 (6.1)	10 (10.2)	24 (8.1)	>0.05
Pulmonary	32 (8.1)	16 (16.3)	16 (5.4)	<0.01‡
Anastomotic leakage	12 (3.0)	3 (3.1)	9 (3.0)	>0.05
Thrombosis	13 (3.3)	6 (6.1)	7 (2.4)	>0.05
Bowel obstruction	11 (2.8)	2 (2.0)	9 (3.0)	>0.05
Postoperative bleeding	13 (3.3)	5 (5.1)	8 (2.7)	>0.05
Gastroparesis	4 (1.0)	1 (1.0)	3 (1.0)	>0.05
Hepatic	4 (1.0)	3 (3.1)	1 (0.3)	<0.05‡
Lymphorrhagia	4 (1.0)	0 (0.0)	3 (1.0)	>0.05
Renal	2 (0.5)	1 (1.0)	1 (0.3)	>0.05
Heart	2 (0.5)	0 (0.0)	2 (0.7)	>0.05
Hypoalbuminemia	10 (2.5)	2 (2.0)	8 (2.7)	>0.05
Others*	8 (2.0)	3 (3.1)	5 (1.7)	>0.05
Death	5 (1.3)	3 (3.1)	2 (0.7)	>0.05

*Values are number of each complication and percent unless indicated otherwise*;

* *Others contain one severe complication (Abdominal pseudocyst formation) and seven mild complications (depression, skin allergies, oral herpes, thrush, delirium, hiccups and acute gout attack.)*;

‡ *Values are statistically significant (P < 0.05); The chi-square test is used for P-values*.

### Univariate and Multivariate Logistic Regression Analyses for Postoperative Complications (PCs)

On univariate analysis, age (*P* = 0.004), NRS score (*P* = 0.057), Charlson comorbidity index (*P* = 0.037), diabetes mellitus (*P* = 0.071), combined resection (*P* = 0.002), laparoscopy-assisted (*P* = 0.010), and DROSD (*P* = 0.013) differed significantly (Table 3). Significant variables on univariate analysis were included in the multivariate logistic regression analysis. Age (OR = 2.459, *P* = 0.014), Combined resection (OR = 3.495, *P* = 0.004), laparoscopy-assisted (OR = 0.222, *P* = 0.044), and DROSD (OR = 2.390, *P* = 0.014) were independently associated with SPCs.

**Table 3:** Univariate and multivariate analysis associated with severe post-operative complications.

**Factors**	**Univariate analysis**			**Multivariate analysis**	
	**Non-SPC (*n* = 353)**	**SPC (*n* = 42)**	***P*-values**	**OR (95% CI)**	***P*-values**
Age, years			0.004‡	2.459 (1.200–5.040)	0.014‡
< 75	292 (82.7)	27 (67.5)			
≥75	61 (17.3)	15 (32.5)			
Gender			0.669		
Female	82 (23.2)	11 (26.2)			
Male	271 (76.8)	31 (73.8)			
BMI, kg/cm2			0.703		
<25	294 (83.3)	34 (81.0)			
≥25	59 (16.7)	8 (19.0)			
NRS score			0.057		
1–2	218 (61.8)	24 (57.1)			
3–4	113 (32.0)	9 (21.4)			
5–6	22 (6.2)	9 (21.5)			
Hypoalbuminemia, g/L			0.306		
No (<30)	343 (97.2)	39 (92.9)			
Yes (≥30)	10 (2.8)	3 (7.1)			
Anemia			0.848		
No	290 (82.2)	34 (81.0)			
Yes	63 (17.8)	8 (19.0)			
PLR			0.609		
≤ 92.8	39 (11.0)	3 (7.1)			
>92.8	314 (89.0)	39 (92.9)			
NLR			0.112		
≤ 2.75	229 (64.9)	22 (52.4)			
>2.75	124 (35.1)	20 (47.6)			
CCI			0.037‡		
0	188 (53.3)	15 (35.7)			
1–2	154 (43.6)	25 (59.5)			
3–6	11 (3.1)	2 (4.8)			
ASA score			0.129		
1–2	287 (81.3)	30 (71.4)			
3–4	66 (18.7)	12 (28.6)			
Hypertension			0.398		
No	265 (75.1)	29 (69.0)			
Yes	88 (24.9)	13 (31.0)			
Diabetes Mellitus			0.071‡		
No	312 (88.4)	33 (78.6)			
Yes	41 (11.6)	9 (21.4)			
Tumor size, cm			0.380		
<4.75	218 (61.8)	23 (54.8)			
≥4.75	135 (38.2)	19 (45.2)			
TNM stages			0.159		
I, II	175 (49.6)	16 (38.1)			
III	178 (50.4)	26 (61.9)			
Total gastrectomy			0.360		
No	219 (62.0)	23 (54.8)			
Yes	134 (38.0)	19 (45.2)			
Combined resection			0.002‡	3.495 (1.504–8.123)	0.004‡
No	326 (92.4)	32 (76.2)			
Yes	27 (7.6)	10 (23.8)			
Laparoscopy-assisted surgery			0.010‡	0.222 (0.051–0.960)	0.044‡
No	277 (78.5)	40 (95.2)			
Yes	76 (21.5)	2 (4.8)			
DROSD			0.013‡	2.390 (1.197–4.772)	0.014‡
No	272 (77.1)	25 (59.5)			
Yes	81 (22.9)	17 (40.5)			

*SPC, severe postoperative complications; BMI, body mass index; PLR, platelet/lymphocyte ratio; NLR, neutrophil/lymphocyte ratio; ASA, American Society of Anesthesiologists; Charlson comorbidity index, CCI; NRS, nutritional risk screening; TNM, tumor-node-metastasis*.

*Values are number of patients and percent unless indicated otherwise*;

‡ *Values are statistically significant (P < 0.05)*.

Based on above results, we also evaluate the relationship between DORSD and postoperative infection complications. On univariate analysis, hypoalbuminemia (*P* = 0.082), NLR (*P* = 0.002), charlson comorbidity index (*P* < 0.001), ASA grade (*P* = *0.031*), tumor size (*P* = 0.003), TNM stages (*P* = 0.004), total gastrectomy (*P* = 0.006), combined resection (*P* = 0.063), laparoscopy-assisted (*P* = 0.004) and DROSD (*P* = 0.018) differed significantly (Table 4). On multivariate analysis, NLR (OR = 1.880, *P* = 0.040), charlson comorbidity index (OR = 2.457, *P* = 0.001), tumor size (OR = 2.105, *P* = 0.016) and laparoscopy-assisted (OR = 0.264, *P* = 0.031) were independently associated with postoperative infectious complications (all *P* < *0.05*).

**Table 4:** Univariate1 and multivariate analysis associated with post-operative infection complications.

**Factors**	**Univariate analysis**			**Multivariate analysis**	
	**Non-PIC (*n* = 339)**	**PIC (*n* = 56)**	***P*-values**	**OR (95% CI)**	***P*-values**
Age, years			0.654		
<75	275 (81.1)	44 (78.6)			
≥75	64 (18.9)	12 (21.4)			
Gender					
Female	82 (24.2)	11 (19.6)	0.458		
Male	257 (75.8)	45 (80.4)			
BMI, kg/cm^2^			0.336		
<25	284 (83.8)	44 (78.6)			
≥25	55 (16.2)	12 (21.4)			
NRS score			0.665		
1–2	209 (61.7)	33 (58.9)			
3–4	104 (30.7)	18 (32.1)			
5–6	26 (7.6)	5 (9.0)			
Hypoalbuminemia, g/L			0.082		
No (<30)	330 (97.3)	52 (92.9)			
Yes (≥30)	9 (2.7)	4 (7.1)			
Anemia			0.467		
No	280 (82.6)	44 (78.6)			
Yes	59 (17.4)	12 (21.4)			
PLR			0.655		
≤ 92.8	37 (10.9)	5 (8.9)			
>92.8	302 (89.1)	51 (91.1)			
NLR			0.002‡	1.880 (1.028–3.439)	0.040‡
≤ 2.75	226 (66.7)	25 (44.6)			
>2.75	113 (33.3)	31 (55.4)			
CCI			<0.001‡	2.457 (1.452–4.519)	0.001‡
0	188 (55.5)	15 (26.8)			
1–2	142 (41.9)	37 (66.1)			
3–6	9 (2.6)	4 (7.1)			
ASA score			0.031‡		
1–2	278 (82.0)	39 (69.6)			
3–4	61 (18.0)	17 (30.4)			
Hypertension			0.122		
No	257 (75.8)	37 (66.1)			
Yes	82 (24.2)	19 (33.9)			
Diabetes Mellitus			0.207		
No	299 (88.2)	46 (82.1)			
Yes	40 (11.8)	10 (17.9)			
Tumor size, cm			0.003‡	2.105 (1.147–3.860)	0.016‡
<4.75	217 (64.0)	24 (42.9)			
≥4.75	122 (36.0)	32 (57.1)			
TNM stages			0.004‡		
I, II	174 (51.3)	17 (30.3)			
III	165 (48.7)	39 (69.7)			
Total gastrectomy			0.006‡		
No	217 (64.0)	25 (44.6)			
Yes	122 (36.0)	31 (55.4)			
Combined resection			0.063		
No	311 (91.7)	47 (83.9)			
Yes	28 (8.3)	9 (16.1)			
Laparoscopy-assisted surgery			0.004‡	0.264 (0.079–0.887)	0.031‡
No	264 (77.9)	53 (94.6)			
Yes	75 (22.1)	3 (5.4)			
DROSD			0.018‡	1.693 (0.891–3.219)	0.108
No	262 (77.3)	35 (62.5)			
Yes	77 (22.7)	21 (37.5)			

*PIC, postoperative infection complications; BMI, body mass index; PLR, platelet/lymphocyte ratio; NLR, neutrophil/lymphocyte ratio; ASA, American Society of Anesthesiologists; Charlson comorbidity index, CCI; NRS, nutritional risk screening; TNM, tumor-node-metastasis*.

*Values are number of patients and percent unless indicated otherwise*;

‡ *Values are statistically significant (P < 0.05)*.

### DROSD and Overall Survival (OS) Rate

We excluded 4 patients who died within 1 month after surgery in order to better study the relationship between DROSD and OS. The remaining 391 patients were included in the analysis. The median follow-up duration was 39.2 months (range, 18.3–45.5 months). At the last follow-up, 151 (38.6%) patients died.

**Figure 3:**
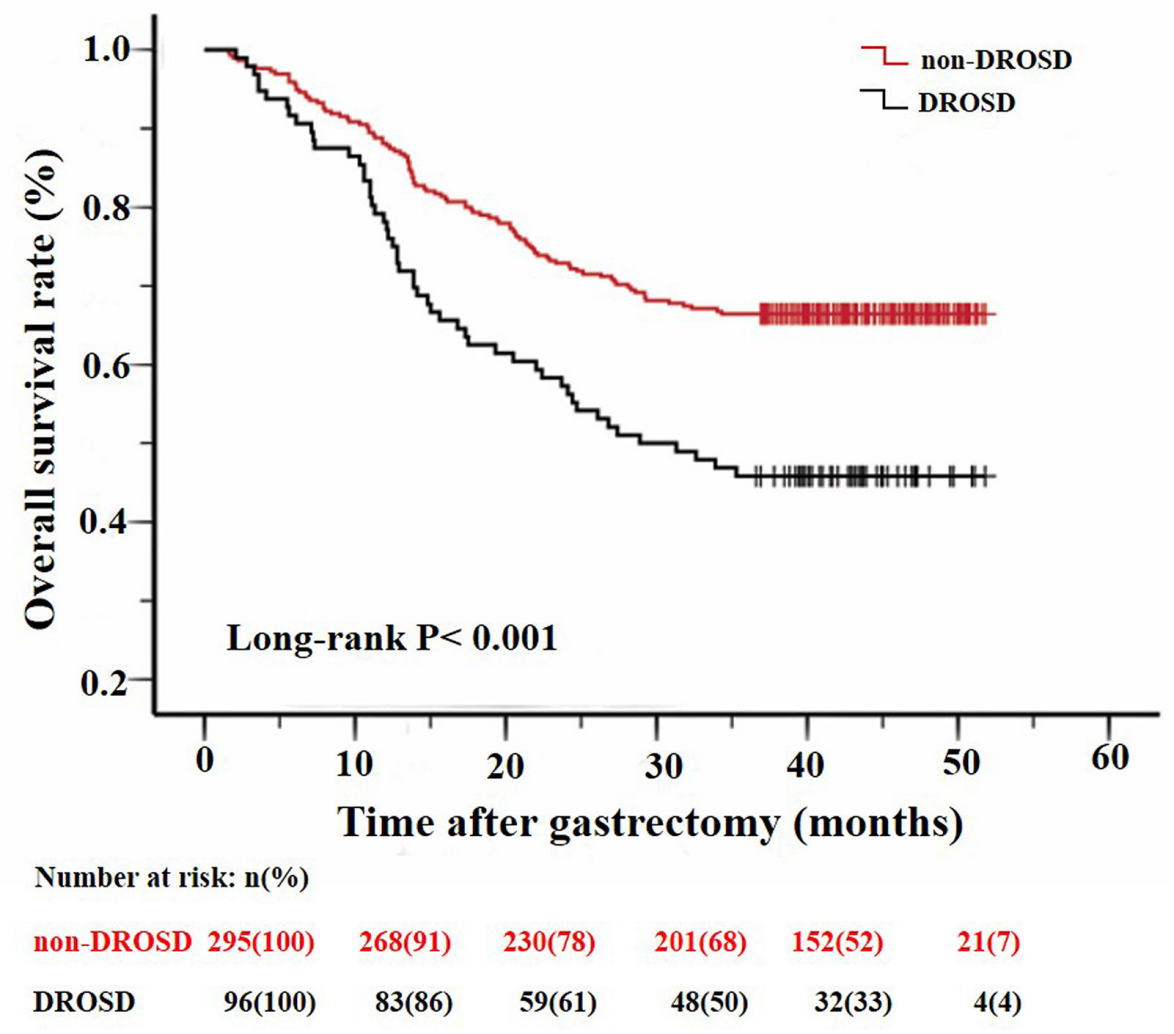
Kaplan–Meier survival curves for overall survival in patients with and in those without DROSD. Two curves were compared using log-rank test.

As shown in Figure 3, patients with DROSD had a poorer OS rate than those without DROSD (*P* < 0.001). The 1- and 3-year overall survival rates were 76.0 and 45.8%, respectively, for patients with DROSD, and were 87.8 and 66.4%, respectively, for those without DROSD. The median OS was shorter in patients with DROSD than in those without DROSD (28.9 vs. 51.7 months; *P* < 0.001; Figure 3).

**Figure 4:**
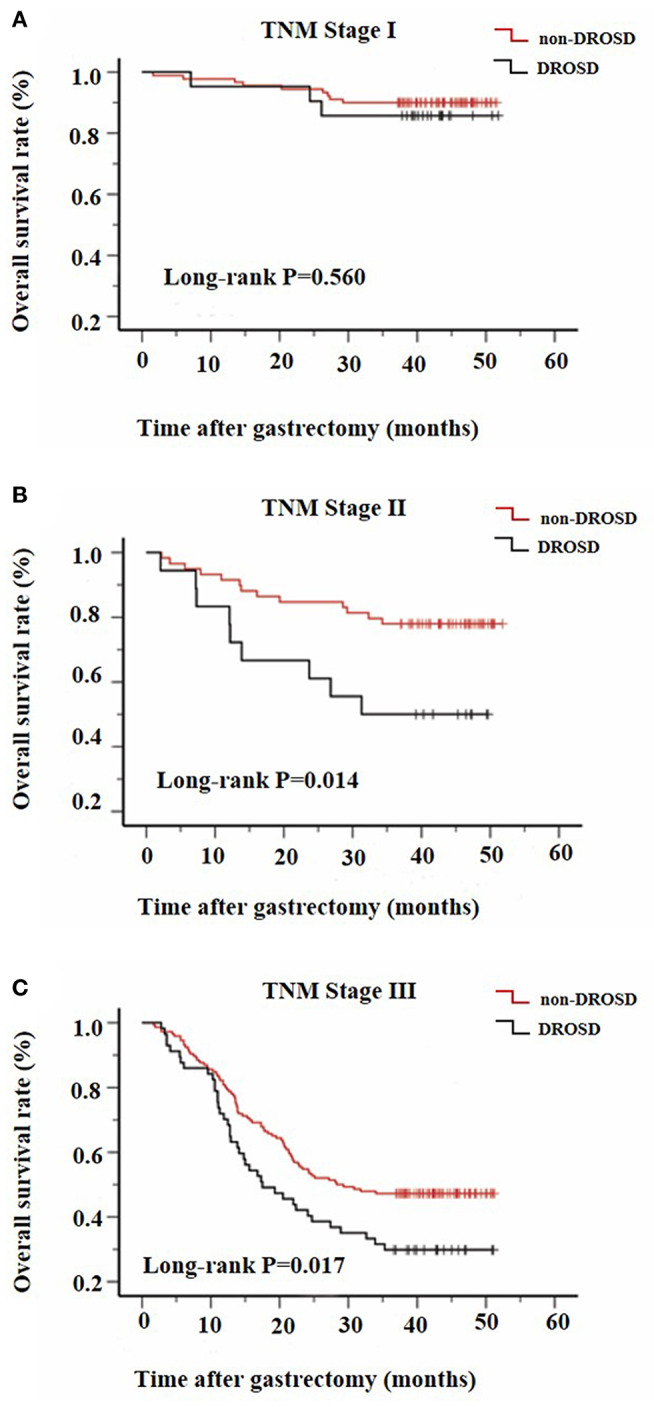
Kaplan–Meier survival curves for overall survival in patients with and in those without DROSD under adjusted TNM stage. Overall survival of patients with **(A)** TNM stage I; **(B)** TNM stage II; and **(C)** TNM stage III gastric cancer.

We further assessed the prognostic value of DROSD in different TNM stage groups. The results revealed that preoperative spleen density was a prognostic indicator in patients with stage II (*P* = 0.014; Figure 4A) and stage III (*P* = 0.017; Figure 4C) gastric cancer. However, for patients with stage I gastric cancer, no significant association of spleen density with OS was identified (*P* = 0.560; Figure 4B).

### Univariate and Multivariate Cox Regression Analysis for OS Rate

As shown in Table 5, in a univariate analysis, age (*P* < 0.0001), NRS 2002 scores (*P* < 0.0001), hypoalbuminemia (*P* = 0.001), NLR (*P* = 0.008), Charlson Comorbidity Index (*P* < 0.0001), ASA grade (P = 0.057), hypertension (*P* = 0.012), differentiation of tumor (*P* < 0.0001), tumor location (*P* < 0.0001), tumor size (*P* < 0.0001), TNM stage (*P* < 0.0001), total gastrectomy (*P* < 0.0001), combined resection (*P* < 0.0001), laparoscopy-assisted surgery (*P* = *0.02*), type of reconstruction (*P* < 0.0001), C–D Classification (*P* < 0.0001), readmission within 30 days (*P* < 0.016), and DROSD (*P* < *0.0001*) were significant prognostic factors.

**Table 5:** Univariate and multivariate analysis of factors associated with overall survival.

**Factors**	**Univariate analysis**		**Multivariate analysis**	
	**HR (95 % CI)**	***P*-values**	**HR (95 % CI)**	***P*-values**
Age, years				
≥75/ <75	1.987 (1.391–2.838)	<0.0001^‡^	1.782 (1.218–2.608)	0.003^‡^
Gender				
Male/female	1.321 (0.886–1.971)	0.172		
BMI, kg/m2				
18.5–23.5/<18.5	0.730 (0.428–1.246)	0.249		
>23.5/<18.5	0.676 (0.385–1.185)	0.171		
NRS 2002 scores				
3–4/1–2	1.369 (0.962–1.948)	0.081		
5–6/1–2	2.721 (1.666–4.444)	<0.0001^‡^		
Hypoalbuminemia				
Yes/No	3.061 (1.558–6.015)	0.001^‡^		
Anemia				
Yes/No	1.176 (0.789–1.754)	0.426		
PLR				
>92.8/≤92.8	1.129 (0.662–1.924)	0.657		
NLR				
>2.75/≤2.75	1.545 (1.120–2.132)	0.008^‡^		
Charlson comorbidity index				
1–2/0	1.676 (1.201–2.340)	0.002^‡^	1.390 (0.980–1.974)	0.064
3–6/0	3.607 (1.845–7. 051)	<0.0001^‡^	3.210 (1.600–6.400)	0.001^‡^
ASA grade				
3–4/1–2	1.433 (0.989–2.077)	0.057^‡^		
Hypertension				
Yes/No	1.551 (1.101–2.185)	0.012^‡^		
Diabetes Mellitus				
Yes/No	1.034 (0.640–1.673)	0.890		
Previous abdominal surgery				
Yes/No	1.330 (0.860–2.058)	0.200		
Differentiation of tumor				
Undifferentiated/Differentiated	2.463 (1.512–4.011)	<0.0001^‡^		
Signet–ring cell/Differentiated	0.783 (0.491–1.248)	0.302		
Tumor location				
Gastric body/cardia	0.818 (0.465–1.441)	0.487	1.001 (0.558–1.797)	0.996
Antrum/cardia	0.757 (0.470–1.219)	0.251	1.593 (0.903–2.810)	0.108
Diffuse/cardia	4.791 (2.451–9.365)	<0.0001^‡^	3.470 (1.690–7.127)	0.001^‡^
Tumor size, cm				
≥4.75/<4.75	2.681 (1.940–3.706)	<0.0001^‡^		
TNM stage				
II/I	2.978 (1.474–6.018)	0.002^‡^	2.780 (1.349–5.731)	0.006^‡^
III/I	7.555 (4.166–13.702)	<0.0001^‡^	6.548 (3.561–12.039)	<0.0001^‡^
Total gastrectomy				
Yes/No	2.629 (1.904–3.631)	<0.0001^‡^	2.433 (1.622–3.650)	<0.0001^‡^
Combined resection				
Yes/no	2.394 (1.534–3.735)	<0.0001^‡^		
Laparoscopy-assisted surgery				
Yes/no	0.579 (0.365–0.918)	0.020^‡^		
Type of reconstruction				
Billroth I/Roux-en-Y	0.277 (0.181–0.423)	<0.0001^‡^		
Billroth II/Roux-en-Y	0.752 (0.502–1.125)	0.165		
C-D complication classification				
≥2/≤1	2.230 (1.590–3.127)	<0.0001^‡^		
Readmission of 30 days				
Yes/No	1.888 (1.125–3.171)	0.016^‡^	2.078 (1.206–3.583)	0.008^‡^
Adjuvant chemotherapy				
Yes/No	1.111 (0.806–1.532)	0.519		
DROSD				
Yes/No	1.897 (1.355–2.654)	<0.0001^‡^	1.568 (1.106–2.223)	0.011^‡^

*BMI, body mass index; NRS, nutritional risk screening; PLR, platelet/lymphocyte ratio; NLR, neutrophil/lymphocyte ratio; ASA, American Society of Anaesthesiologists; diffuse, linitis plastic; TNM, tumor-node-metastasis, C-D complication classification, Clavien-Dindo complication classification; HR, hazards ratio; CI, confidence interval*.

*4 patients who died within 1 month after surgery was excluded in order to better study the relationship between DROSD and overall survival. The remaining 391 patients were included in the analysis*.

‡ *values are statistically significant (P < 0.05)*.

Significant variables in a univariate analysis were included in multivariate Cox regression analysis. We found that age (HR = 1.782, *P* = 0.003), Charlson Comorbidity Index of 3–6 (HR = 3.210, *P* = 0.001), diffuse gastric cancer (HR = 3.470, P = 0.001), advanced TNM stage (P < 0.0001), total gastric resection (HR = 2.433, *P* < 0.0001), readmission within 30 days (HR = 2.078, *P* = 0.008), and DROSD (HR = 1.568, *P* = 0.011) were independently associated with a lower OS rate.

## Discussion

The topic of post-operative outcomes after curative gastrectomy is of great concern for both surgeons and patients (14, 15). To study the related factors affecting prognosis of gastric cancer and early identification of patients with a poor prognosis will be particularly meaningful (16). There are many indicators to predict the prognosis of gastric cancer (17, 18). The downside of TNM stage, a classic indicator of prognosis for gastric cancer, was determined postoperatively (19). Currently, lack of efficient means to early predict postoperative outcomes of patients has been considered as one of the obstacles for improve prognosis of gastric cancer.

In contrast, as an imaging evaluation method, the measurement of spleen density by CT is compatible with daily clinical practice because it is well-visualized, cost-effective, and can easily be diagnosed preoperatively. CT of the abdomen is currently the primary means of staging for gastric cancer and is widely available in clinical practice (20). However, the value of abdominal CT has not been fully reflected. Spleen density is a novel tool that can be used in clinical practices as a prognostic predictor for patients with gastric cancer. In the present study, we reported that the incidence rate of DROSD was up to 24.8% in the patient cohort. Furthermore, DROSD was identified as an independent risk factor for post-operative complications (OR = 2.390, *P* = 0.014) and long-term OS (HR = 1.568, *P* = 0.011) in gastric cancer. Through this paper, we hope that patients with poor prognosis can be screened early according to spleen density.

This is the first time we reported that DROSD has a negative impact on short- and long-term prognosis for patient after radical gastrectomy. The mechanisms by which DROSD confers increased risk of poor prognosis are still unclear, but the following reasons can be hypothesized. First, spleen is an abdominal parenchymal organ of the body (21). AP patients with DROSD have downregulated immune function, and DROSD patients had more severe lymphocytes decreased than non-DROSD patients (7). It is worth noting that immune function were associated with favorable prognosis for gastric cancer patients (22–24). Meantime, we also found that DROSD patients have a higher NLR level (*P* < 0.05). NLR is an indicator of systemic inflammatory response (25). The higher NLR level can enhance the occurrence of inflammatory cytokine cascades (26) and negatively affect the immune system, which can partially explain the negative impact of DROSD on post-operative outcomes. Whether gastric cancer patients with DROSD have also experienced the function disorder of immunity, which lead to the patient having short- and long-term differences still requires further experimental verification. Second, it has been reported that splenic volume increase is a surrogate marker of inflammation cells accumulation and associated with worse long-term survival (27, 28). As mentioned in the previous literature, the reduction of spleen density in acute severe pancreatitis rats was related to the increase of spleen volume (7). There may be a correlation between the spleen density and the volume in human, which leads to spleen density associated with poor long-term prognosis. Third, some scholars have speculated that DROSD is caused by spleen fat infiltration (6). Does obesity affect the prognosis of cancer patients? Visceral fat area, as an evaluation index of obesity, can evaluate operative difficulties and is reportedly associated with post-operative complications (29, 30). However, BMI has little to do with long-term prognosis in the present study (*P* > 0.05). Even the previous literature has pointed out that obesity is a protective factor for the long-term prognosis of cancer patients (31–33).

Spleen density is susceptible to many factors, such as hemoperfusion. The mechanism for the DROSD is not fully expounded. It has been reported that spleen density reduction is associated with lipid metabolism (6). Some animal experiments show that DROSD is not related to lipid deposition but hemoperfusion (7). The rich blood flow of spleen can change physical density, thereby affecting its density value on CT (34, 35). This is consistent with the result in Table 1, that is, DROSD is associated with hypertension and preoperative hemoglobin concentration (*P* < 0.05). Whether DROSD is related to lipid metabolism or hemoperfusion needs to be further studied in animal and human studies. Meantime, we also noticed that NLR and others, as possible confounding factors of spleen density, can affecting the reliability of conclusion. Therefore, factors such as NLR and hypoalbuminemia were included in the multivariate analysis, and the results showed that DROSD was an independent risk factor that affected the post-operative outcome of gastric cancer patients.

We found that old age, charlson comorbidity index of 3–6, diffuse gastric cancer, advanced TNM stage, total gastric resection, and readmission within 30 days were associated with poor prognosis in patients with gastric cancer. The survival difference in the elderly patients and young patients can be partially explained by the dissimilarity in treatment (36). Liu et al. (37) found that the OS rate after distal gastrectomy for distal gastric cancer patients was higher than that after total gastrectomy. Moreover, some literature also pointed out that Charlson Comorbidity Index, diffuse gastric cancer, and readmission were also associated with poor long-term survival (38–40).

Similar to our research result, tumor staging is closely related to long-term prognosis of cancer (17). To objectively evaluate the impact of spleen density on the OS, we stratified the patients according to their TNM stage. The results showed that DROSD group has a significantly poorer OS than the non-DROSD group, under TNM stage II and III (*P* < 0.05). However, for patients with TNM stage I, the difference was not significant, although there was a trend toward worse OS in the DROSD patients. Since patients with TNM stage I generally have a longer postoperative survival time, we propose that a longer follow-up period is needed to further research the effect of DROSD on long-term postoperative survival.

Our study had several potential limitations that should not be ignored. First, the data of this study were obtained only from double hospital, and a bias may exist due to the lack of multicenter validation of the research conclusions. Second, even we assigned two investigators to measure the spleen density together, the artificial measurement errors still exist.

## Conclusions

DROSD is an independently risk factor for severe postoperative complications and long-term overall survival in gastric cancer patients. As an imaging evaluation method, spleen density is a novel tool can be used in clinical as a prognostic predictor for patients with gastric cancer.

## Data Availability Statement

The datasets generated for this study are available on request to the corresponding author.

## Ethics Statement

This retrospective study was approved by the Ethics Committee of the First Affiliated Hospital of Wenzhou Medical University and the Second Affiliated Hospital of Wenzhou Medical University.

## Author Contributions

All authors contributed in drafting the manuscript and revising it critically. Furthermore, they were involved in the following tasks. Y-SH planned and designed the study and directed its implementation. X-DC drafted the protocol. M-MS obtained statutory and ethics approvals. L-BX contributed to data acquisition. S-JW conducted statistical analyses. W-SC had access to all raw data. XS did the data preparation and quality control. W-TZ and G-BZ wrote and revised the manuscript. All authors read and approved the final manuscript prior to submission.

## Conflict of Interest

The authors declare that the research was conducted in the absence of any commercial or financial relationships that could be construed as a potential conflict of interest.
