# Supplementary figures and images for: Diffuse Reduction of Spleen Density Is an Independent Predictor of Post-Operative Outcomes After Curative Gastrectomy in Gastric Cancer: A Multi-Center Study
Source: Front Oncol. 2020 Jun 30;10:1050. doi: 10.3389/fonc.2020.01050 (PMC7340088; doi:10.3389/fonc.2020.01050)

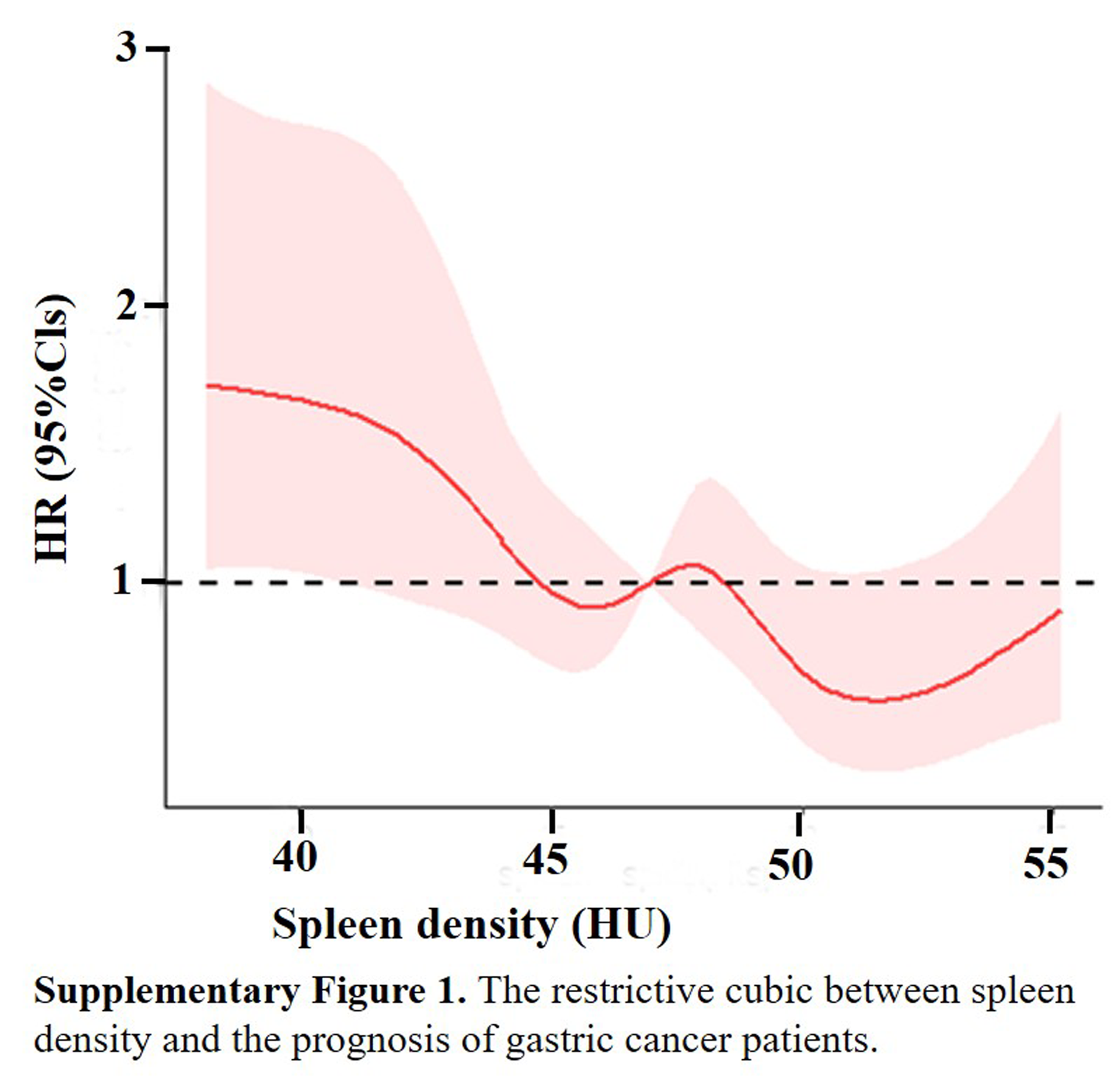

Supplement: Supplementary file 1 [file Image_1.TIF]
